# Extreme Fluid Accumulation Syndrome or Compartmental Balance Disorder? A Sepsis-Associated Acute Kidney Injury Case Report and Literature Review

**DOI:** 10.3390/jcm14238310

**Published:** 2025-11-22

**Authors:** Michael Cieza Terrones, Celia Rodríguez Tudero, Avinash Chandu Nanwani, Elena Jiménez Mayor, Marco Dominguez Davalos, José C. De La Flor, Misael Cieza Armas, Gregorio Romero-González, Jonathan S. Chávez-Iñiguez

**Affiliations:** 1 Department of Engineering, Faculty of Science and Engineering, Peruana Cayetano Heredia University, Lima 15012, Peru; michael.cieza@upch.pe (M.C.T.); misael.cieza@upch.pe (M.C.A.); 2 Department of Nephrology, Clínica Anglo Americana, Lima 15073, Peru; 3 Department of Nephrology, Hospital Universitario de Salamanca, 37007 Salamanca, Spain; crodrigueztudero@usal.es; 4 Surgery Department, Faculty of Medicine, University of Salamanca, 37007 Salamanca, Spain; 5 Department of Nephrology, Hospital General de Fuerteventura, 35600 Fuerteventura, Spain; achanan@gobiernodecanarias.org; 6 Department of Nephrology, Hospital Santa Bárbara, 42003 Soria, Spain; ejimenezmay@saludcastillayleon.es; 7 Department of Nephrology, Hospital Cayetano Heredia, Lima 15002, Peru; marco.dominguez.d@upch.pe; 8 Department of Nephrology, Hospital Central de la Defensa Gomez Ulla, 28047 Madrid, Spain; 9 Department of Medicine and Medical Specialties, Faculty of Medicine, Alcala University, 28805 Madrid, Spain; 10 Health Sciences Doctoral Program, Faculty of Medicine, Alcala University, 28805 Madrid, Spain; 11 Grupo REcerca en Malalties d’Afectació Renal (REMAR)-Investigación Germans Trias i Pujol (IGTP), Department of Nephrology, Hospital Germans Trias i Pujol, 08916 Badalona, Spain; garomerog.germanstrias@gencat.cat; 12 Department of Nephrology, Hospital Civil de Guadalajara Fray Antonio Alcalde, Guadalajara 44200, Mexico; jschavez@hcg.gob.mx; 13 Department of Medicine and Health Sciences, Faculty of Medicine, University of Guadalajara, Guadalajara 44100, Mexico

**Keywords:** fluid accumulation syndrome, venous excess ultrasound score, sepsis associated acute kidney disease, bioelectrical impedance analysis, compartment balance disorder in intensive care units

## Abstract

**Background:** Fluid accumulation syndrome (FAS) is a well-recognized predictor of adverse outcomes in critically ill patients, particularly in the context of sepsis and cardiorenal syndrome. However, extreme cases of fluid accumulation exceeding 60 L are rare and poorly described. We report a unique case of severe, multifactorial congestion and discuss the diagnostic and therapeutic challenges, including the role of bedside ultrasound and venous congestion assessment, as well as the importance of bioelectric impedance analysis (BIA) for patient monitoring and follow-up. **Case Presentation:** We describe the clinical course of a 51-year-old male with dilated cardiomyopathy and infectious endocarditis who underwent tricuspid valve surgery complicated by cardiogenic and septic shock. The patient developed progressive congestion despite maximal medical management. Serial BIA and venous excess ultrasound (VExUS) assessments were used to monitor venous congestion and guide renal replacement therapy (RRT). A targeted literature review was performed to contextualize this case within current evidence on FAS and Compartment Balance Disorder in Intensive Care Units (CBD-ICUs). **Results:** The patient accumulated over 68 L of positive fluid balance due to prolonged vasopressor support, multiorgan failure, nutritional and infectious complications. Continuous and then intermittent hemodiafiltration, guided by point-of-care ultrasound and BIA, allowed gradual decongestion. Renal function recovered, and ventilator support was weaned after 120 days in intensive care. The literature review highlighted the prognostic relevance of FAS and emerging tools such as BIA and VExUS for individualized fluid management. **Conclusions:** Extreme FAS may reflect a failure of systemic and compartmental fluid regulation in critically ill patients (CBD-ICU). VExUS-guided decongestion, BIA and early RRT may improve outcomes in complex scenarios of overlapping cardiorenal and septic syndromes.

## 1. Introduction

Sepsis remains a leading cause of admission and mortality in intensive care units (ICUs), with fluid resuscitation forming a cornerstone of initial management strategies [1]. In the early phases of septic shock, prompt fluid administration restores intravascular volume, augments cardiac output, and improves tissue perfusion and oxygen delivery. However, fluid therapy is a double-edged sword: while essential for hemodynamic stabilization, excessive or prolonged administration predisposes patients to fluid overload (FO), which is consistently associated with adverse outcomes, including sepsis-associated acute kidney injury (SA-AKI), prolonged mechanical ventilation (VM), increased length of hospital stay, and higher mortality. Notably, even moderate congestion has been linked to poor prognosis, underscoring the need for precise fluid assessment beyond the resuscitation phase [2].

Fluid accumulation syndrome (FAS) is increasingly recognized as a distinct clinical entity in critical care. Its pathophysiology is multifactorial, driven by systemic inflammation, endothelial glycocalyx degradation, increased vascular permeability, and venous congestion [2]. The resulting capillary leak and redistribution of fluids between compartments disrupt intravascular–interstitial dynamics, complicating fluid assessment and management. Organ congestion, particularly renal venous congestion, further amplifies the deleterious effects of FAS by impairing kidney perfusion, perpetuating oliguria, and creating a vicious cycle in which fluid is administered in response to low urine output, thereby worsening overload [3]. Most organ systems—including the lungs, heart, and gastrointestinal tract—are adversely affected by FAS, highlighting its systemic impact [2].

Despite the clinical relevance of FAS, monitoring of fluid balance remains imprecise. Cumulative fluid balance (CFB) calculations, physical examination findings (edema, jugular venous distension), and central venous pressure (CVP) are widely employed but lack sensitivity and specificity [4]. These tools fail to differentiate between intra- and extravascular compartments and are prone to significant interobserver variability. Chest radiography, traditionally used to assess pulmonary congestion, offers only indirect and non-specific markers of hypervolemia. More advanced hemodynamic monitoring techniques, such as transpulmonary thermodilution, provide information on extravascular lung water and pulmonary vascular permeability but are invasive, costly, and not universally available. Consequently, there is no universally accepted gold standard for FO assessment in critically ill patients [2].

In recent years, non-invasive technologies have emerged as promising alternatives. Bioelectrical impedance analysis (BIA) has gained attention as a reproducible bedside tool capable of estimating total body water (TBW), extracellular water (ECW), intracellular water (ICW), overhydration (OH), and derived indices such as the ECW/ICW ratio and phase angle [5].

BIA relies on measuring tissue resistance and reactance to a low-amplitude electrical current and has been validated against reference methods in chronic kidney disease (CKD), hemodialysis, and heart failure populations [6]. While its application in the ICU is less established, preliminary studies suggest that BIA can detect early FO, monitor intercompartmental fluid shifts, and potentially guide fluid removal strategies [6]. Its advantages include being non-invasive, rapid, inexpensive, and relatively easy to perform with minimal training. An additional advantage is that it allows you to define the edematous states generated by water overload in intensive care units, which we will call Compartment Balance Disorder (CBD) in ICU, where the large water supply generates an increase in the aqueous compartment (intracellular, interstitial and intravascular) that leads to an abnormal increase in the Fat-Free Mass (FFM) and therefore to a dysfunction of the biological membranes [7].

Ultrasound-based modalities complement BIA by providing compartment-specific insights. Lung ultrasound, widely available at the bedside, enables detection and semi-quantification of extravascular lung water via B-lines and has demonstrated prognostic value in both heart failure and sepsis [8]. Vascular ultrasound of the inferior vena cava (IVC) or internal jugular vein can estimate right atrial pressure and aid in assessing fluid responsiveness, though its reliability may be limited by MV and intra-abdominal pressures [9,10].

The integration of BIA with targeted ultrasound represents a multimodal, physiology-based strategy to overcome the limitations of conventional monitoring. By combining whole-body composition analysis with localized imaging, clinicians can better identify early FO, characterize intercompartmental shifts, and personalize fluid management in high-risk patients. Recent conceptual models, such as the SOSD (salvage, optimisation, stabilisation, and de-escalation) and the ROSE (resuscitation, optimisation, stabilisation, evacuation) frameworks, highlight the importance of considering fluids as drugs that must be prescribed according to type, dose, duration, and strategy for withdrawal [2]. Despite these advances, most trials have been conducted in general ICUs populations, and specific evidence for SA-AKI is lacking, particularly in patients with concomitant cardiogenic dysfunction or extreme FAS. This gap is clinically relevant, as venous congestion and compartmental fluid shifts can significantly compromise renal recovery and overall outcomes.

In this context, we report a unique case of severe, multifactorial FO exceeding 68 L in a patient with septic shock and advanced dilated cardiomyopathy, illustrating the limitations of conventional monitoring and highlighting the potential role of integrating BIA and ultrasound to individualize fluid strategies. Beyond its rarity, this case emphasizes the clinical importance of adopting innovative bedside technologies to optimize volume management, preserve organ function, and potentially improve outcomes in critically ill patients with FAS.

## 2. Case Report

**Figure 1:**
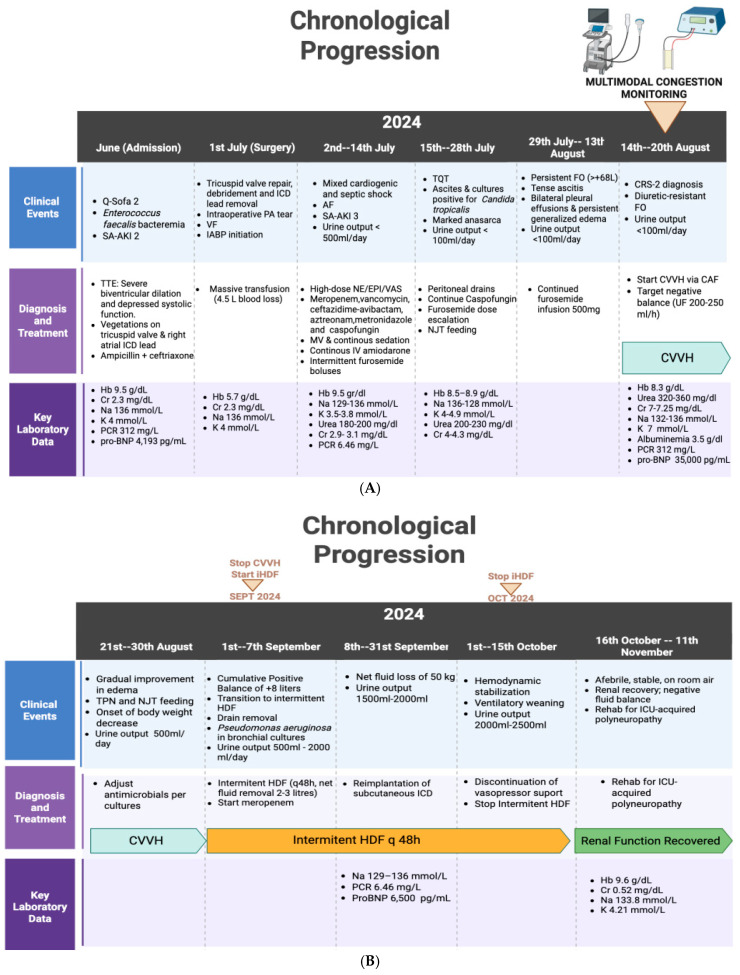
(**A**). Chronological progression of the patient’s clinical course, major interventions, and representative laboratory parameters from June to mid-August 2204. (**B**). Chronological progression of the patient’s clinical course, major interventions, and representative laboratory parameters from mid-August 2024 to mid-November 2024.Values are selected to illustrate significant changes in organ function, fluid status, and systemic inflammation during hospitalization. Created in https://BioRender.com. Abbreviations: Hb: hemoglobin; Cr: creatinine; Na: sodium; K: potassium; PCR: C-reactive protein; pro-BNP: N-terminal pro–B-type natriuretic peptide; PA: pulmonary artery; VF: ventricular fibrillation; IABP: intra-aortic balloon pump; NE: norepinephrine; EPI: epinephrine; VAS: vasopressin; MV: mechanical ventilation; AF: atrial fibrillation; TEE: transesophageal echocardiography; TQT: tracheostomy; NJT: nasojejunal tube; FO: fluid overload; CRS-2: cardiorenal syndrome type 2; CVVH: continuous veno-venous hemofiltration; CAF: central access femoral catheter; TPN: total parenteral nutrition; HDF: hemodiafiltration; SFA: superficial femoral artery; ICD: implantable cardioverter-defibrillator; UF: ultrafiltration.

A 51-year-old male with a medical history of dilated cardiomyopathy (DCM) with reduced left ventricular ejection fraction (LVEF < 35%), implantable cardioverter-defibrillator (ICD), obesity, hypertension (HTN), type 2 diabetes mellitus (T2DM), and previous colon cancer status post-surgery and adjuvant chemotherapy, admission to the Anglo-American Clinic (Lima, Peru) in June 2024, with persistent fever, progressive fatigue, and dyspnea. Subsequent evaluation revealed Enterococcus faecalis bacteremia with large vegetations on the tricuspid valve (31 mm and 18 mm) and on the right atrial ICD lead, confirmed by transesophageal echocardiography (TEE). On arrival, he was febrile (38.7 °C), tachycardic (heart rate 122 bpm), hypotensive (systolic blood pressure < 100 mmHg; mean arterial pressure 58 mmHg), tachypneic (respiratory rate 28 breaths/min), and had a Glasgow Coma Scale (GCS) score of 15 (qSOFA 2). Physical examination revealed jugular venous distension, bilateral lower limb pitting edema, and bibasilar crackles. Laboratory data shown in Figure 1A,B. Chest radiography showed cardiomegaly with prominent pulmonary vasculature and probable right hilar infiltrates, while transthoracic echocardiography (TTE) confirmed severe biventricular dilation and depressed systolic function. Broad-spectrum antimicrobial therapy with intravenous ampicillin (2 g IV every 4 h) and ceftriaxone (2 g IV every 12 h) was initiated, targeting the *Enterococcus faecalis* bacteremia secondary to infective endocarditis.

In July 2024, the patient underwent tricuspid valve repair with posterior leaflet reconstruction, surgical debridement, and removal of the ICD lead. The procedure was complicated by a left pulmonary artery (PA) tear requiring patch repair, prolonged cardiopulmonary bypass, refractory ventricular fibrillation (VF) requiring multiple defibrillations and initiation of intra-aortic balloon pump (IABP) support at a 1:2 ratio. Estimated intraoperative blood loss was 4500 mL, requiring massive transfusion. Postoperatively, he was transferred to the ICU in mixed cardiogenic and septic shock, requiring high-dose norepinephrine (NE up to 0.8 µg/kg/min), epinephrine (EPI up to 0.3 µg/kg/min), and vasopressin (VAS 0.04 U/min), mechanical ventilation (MV), and continuous sedation. Atrial fibrillation (AF) was managed with continuous IV amiodarone. Urine output progressively declined from <500 mL/day in the first days to <100 mL/day by the end of the second postoperative week, despite continuous furosemide infusion, with deterioration of renal function up to serum creatinine (sCr) of 4.3 mg/dL (with normal baseline sCr < 1 mg/dL) consistent with SA-AKI (Figure 1).

Persistent fever prompted repeated blood cultures and removal of invasive lines. Echocardiography confirmed severe biventricular dysfunction, and abdominal ultrasound showed no acute abdominal pathology. Broad-spectrum antimicrobial coverage was escalated to ceftazidime/avibactam (2.5 g/0.5 g IV every 8 h), aztreonam (2 g IV every 8 h), metronidazole (500 mg IV every 8 h), and vancomycin (15 mg/kg IV every 12 h, AUC-guided), with subsequent addition of caspofungin (70 mg IV loading dose followed by 50 mg IV once daily) for suspected candidemia.

In the third week, tracheostomy (TQT) was performed due to prolonged MV requirements. Abdominal computed tomography (CT) revealed abundant ascites. Interventional radiology placed peritoneal drains, with cultures positive for Candida tropicalis, prompting reinitiation of caspofungin at 50 mg IV once daily (after a prior 70 mg IV loading dose). Despite maximal diuretic therapy (240 mg/day), the patient developed marked anasarca, tense ascites, and bilateral pleural effusions. His body weight rose from 92 kg on admission to 160 kg by the end of July, corresponding to a cumulative positive fluid balance exceeding +68 L. Peripheral edema extended to all extremities, and abdominal distension was pronounced.

**Figure 2:**
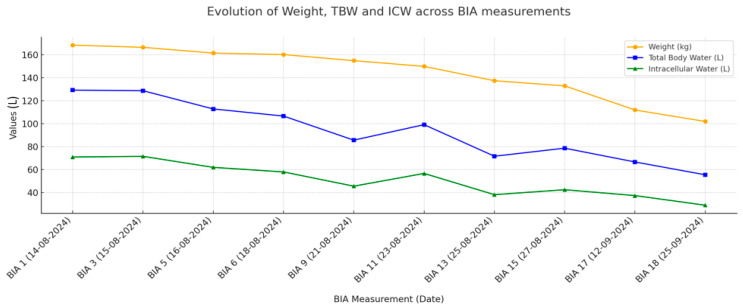
Evolution of weight, total body water (TBW) and intracellular water (ICW) across serial BIA measurements. Abbreviations: BIA, bioelectrical impedance analysis; TBW, total body water; ICW, intracellular water.

**Figure 3:**
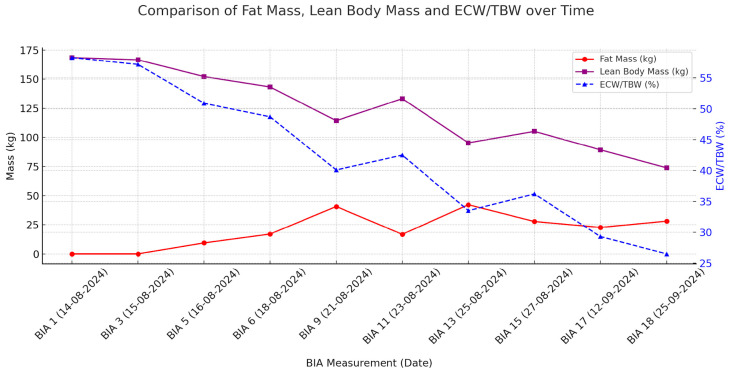
Comparison of fat mass (FM), lean body mass (LBM) and extracellular water/intracellular water (ECW/TBW) during fluid removal. Abbreviations: FM, fat mass; LBM, lean body mass; ECW, extracellular water; TBW, total body water; BIA, bioelectrical impedance analysis.

By mid-August, the patient remained critically ill with persistent generalized edema, tense ascites, and oliguria progressing to anuria despite continuous intravenous furosemide infusion at 20 mg/h (≈480 mg/day), the patient progressed to KDIGO stage 3 AKI (sCr 7–7.25 mg/dL) according to the 2020 KDIGO guidelines [11]. Diuretic resistance was evident, and type 2 cardiorenal syndrome (CRS-2) was diagnosed. Subsequently, continuous veno-venous hemofiltration (CVVH) was initiated via a femoral central access catheter (CAF) due to refractory FO. Ultrafiltration (UF) was commenced cautiously at 200–250 mL/h to avoid hemodynamic compromise and was progressively increased as tolerated. Multimodal congestion monitoring was with bioimpedance analysis (BIA) (medical body composition analyzer, seca mBCA 525) (seca GmbH & Co., KG, Hamburg, Germany), and the venous excess ultrasound (VExUS) protocol. Serial BIA was performed at predefined inpatient timepoints (Table 1, Figure 2 and Figure 3).

From late August, with careful UF and supportive therapy, body weight began to decrease. By the first week of September, weight had reduced to approximately 135 kg, reflecting a net fluid removal of around 25 kg from peak. Edema improved, and spontaneous diuresis resumed, exceeding 500 mL/day by the end of August.

In early September, the patient transitioned from CVVH to intermittent hemodiafiltration (iHDF) every 48 h, each session achieving net fluid removal of 2–3 L. Drains were removed as abdominal collections resolved radiologically. At this time, daily urine output increased to 1.5–2.0 L, and cumulative positive balance decreased to approximately +8 L.

By the end of September, the patient’s body weight had decreased further to approximately 110 kg, representing a net fluid loss of 50 kg from peak overload. Daily urine output was stable at >1.5 L, peripheral edema was minimal, and abdominal girth had decreased significantly. The subcutaneous ICD was reimplanted. Over the course, TBW and ECW declined steadily, with a modest rise in phase angle and an increase in resistance and reactance, consistent with effective decongestion; complete values are summarized in Table 1. These trends are illustrated in Figure 2, which depicts the progressive reduction in weight, TBW and ICW across serial BIA measurements. During the transition from CVVH to iHDF, the ECW/TBW ratio fell from 46–47% to approximately 45%, while reactance increased later in the course, paralleling stepwise improvement in VExUS findings (Table 1), which revealed persistent but improving venous congestion with decreased portal vein pulsatility and normalization of hepatic vein flow patterns. Figure 3 shows the relative trajectories of fat mass, lean body mass and ECW/TBW ratio, highlighting how apparent reductions in lean tissue partly reflect fluid shifts.

By early October, the patient showed sustained hemodynamic stability with minimal vasopressor support, allowing complete discontinuation. Body weight had returned to approximately 94 kg, close to his baseline before hospitalization, representing a net fluid loss of more than 65 kg from peak overload. This correlated with sustained negative fluid balance and a daily urine output consistently above 2 L without the need for UF.

Ventilatory support was progressively weaned from continuous positive airway pressure (CPAP) to tracheostomy collar, and finally to spontaneous breathing in ambient air. Cardiovascular management included the introduction of continuous intravenous dobutamine at approximately 3 µg/kg/min to optimize cardiac output, and discontinuation of the patient’s beta-blocker (bisoprolol 2.5 mg once daily) due to bradycardia and recurrent low-output episodes and discontinuation of beta-blockers due to bradycardia and low-output episodes. Despite underlying DCM with LVEF persistently <35%, the patient maintained adequate systemic perfusion and oxygenation.

Finally, his weight remained stable at 94 kg, with a neutral to slightly negative daily fluid balance, and urine output consistently between 2–2.5 L/day under maintenance diuretic therapy.

## 3. Discussion

We presented a case of FAS and CBD-UCI exceeding 68 L measured by BIA (Table 1) in a critically ill patient with septic and cardiogenic shock complicated by CRS-2. This magnitude is rarely described and exemplifies how overlapping septic–cardiorenal pathophysiology can produce profound systemic and compartmental derangements in fluid regulation. Despite maximal pharmacologic therapy, including high-dose diuretics, the patient developed diuretic resistance and required RRT for progressive congestion. In this scenario, multimodal monitoring with BIA [7], LUS, and VExUS yielded actionable signals that weight, CVP, or isolated laboratory trends could not provide. Serial VExUS guided the timing and intensity of UF, LUS quantified pulmonary congestion, and BIA corroborated whole-body excess. This integrated strategy enabled a safe transition from continuous to intermittent RRT, avoidance of intradialytic hypotension, and recovery of renal function with spontaneous diuresis, aligning with contemporary frameworks for phase-specific fluid stewardship in SA-AKI [12].

Within this context, BIA emerges as a feasible, operator-independent technique estimating TBW, ECW, and ICW, and deriving absolute FO and relative FO to complement balance charts. However, expert commentary emphasizes that BIA is “promising but not yet ready for prime time,” advocating serial, trend-based integration rather than single time-point decisions [4]. In our case, de-resuscitation was operationalized with VExUS and LUS as congestion endpoints, guiding stepwise UF from CVVH to iHDF. BIA would have added quantitative “how-much” targets (absolute/relative FO) to the “where/when” signals provided by VExUS/LUS, particularly during the transition from continuous to intermittent therapy. Normalization of VExUS paralleled the return of spontaneous diuresis and hemodynamic stability, supporting ultrasound-defined decongestion as a pragmatic surrogate endpoint for UF titration in severe FO [4].

Evidence synthesized across BIA-focused cohorts links hyperhydration to worse outcomes (prolonged ventilation, higher mortality) and identifies FO at continuous RRT initiation as a marker of poorer prognosis—reinforcing earlier, biomarker- and imaging-guided decongestion once fluid non-responsiveness is established [4]. Our patient fit that profile: diuretic resistance with escalating venous congestion, prompting UF after which weight decreased from ~160 kg to ~94 kg with recovery of urine output and liberation from VM, a trajectory consistent with congestion-targeted de-resuscitation principles. In parallel, the SA-AKI framework underscores phase-specific stewardship from salvage to de-resuscitation and the need for better tools to identify when intravascular status is adequate so that fluids cease—gaps amplified in mixed septic–cardiogenic physiology [12].

In a single-center randomized trial, BIA-vector analysis (BIVA); guided UF during CRRT with 8-hourly reassessments and 50–100 mL/h increments capped at 250 mL/h yielded lower lean-body-mass water, higher mean UF rates (~1.5 vs. ~1.1 mL/kg/h), and greater urine output during CRRT without excess hypotension or vasopressors, albeit without differences in ICU length of stay, ventilation duration, or mortality [13]. Applying BIVA and VEXUS score to adjust UF rate in critically ill patients on continuous renal replacement therapy, our practice of initiating UF at 200–250 mL/h and transitioning from CVVH to intermittent HDF as congestion improved mirrors this protocolized ceiling and coheres with the trial’s signal toward greater diuresis under device-informed decongestion—pairing BIVA “how much” with VExUS/LUS “where/when” to individualize de-resuscitation in FAS [13].

Beyond the ICU trial context, a 2022 systematic review in heart failure and non-dialysis chronic kidney disease linked bioimpedance-derived FO indices with higher risks of all-cause mortality, cardiovascular events, and CKD progression; the largest CKD cohort (*n* = 3751) showed approximately two-fold higher mortality for lower phase-angle strata and higher risks of HF events and CKD progression. The authors proposed Body Composition Monitor (BCM)-derived refractory FO thresholds of >7% (moderate) and >15% (severe), approximating +1.1 L and +2.5 L of absolute FO, respectively—practical anchors for defining decongestion targets, even though sepsis/critical illness populations were excluded [6]. In our patient, the accumulation (>+68 L) vastly exceeded “severe” relative FO by any plausible ECW estimate, underscoring the rationale to combine bioimpedance “how much” with VExUS/LUS “where/when” to individualize UF endpoints in an extreme FO phenotype [6].

Cohort data in AKI further support whole-body targeting: in men with AKI starting continuous veno-venous hemodiafiltration, multi-frequency BIA (MF-BIA); defined TBW indexed to height squared (TBW/H^2^) independently predicted in-hospital mortality (≈1.31 OR per 1 L/m^2^), and only excess ICW/H^2^—not ECW—remained independently associated with death; mortality increased stepwise across TBW/H^2^ quartiles (~31% to 54%) [14]. In our case, the parallel between VExUS normalization, rising diuresis, and hemodynamic stabilization is congruent with the notion that reducing both venous and intracellular congestion is clinically meaningful; serial MF-BIA could have quantified the ICW trajectory to pair the “how-much” signal with VExUS/LUS “where/when,” while guarding against intradialytic hypotension. A 2024 systematic review focused on kidney disease and volume analysis validates BIA for hydration assessment but highlights the predominance of observational designs and calls for protocol standardization and randomized trials—supporting BIA as an adjunct integrated with imaging/hemodynamics rather than a stand-alone trigger for UF [7].

The FAS construct—any percentage weight increase from baseline plus new organ failure—advocates prevention and stepwise de-resuscitation, limiting sodium/“fluid creep,” early vasopressors to avoid reflex fluid loading, and active fluid removal once non-responsiveness is documented, even under vasopressor support [2]. This is consonant with ICU evidence linking FAS to worse outcomes, the limited value of static markers, and the escalation pathway from diuretics to extracorporeal therapies; CRRT affords hemodynamically gentle UF with typical rates around 100–300 mL/h and precise balance control—parameters mirrored in our 200–250 mL/h strategy and CVVH to iHDF transition [15]. For risk stratification and target-setting at CRRT start, BIA-derived ECW/TBW thresholds (≥0.41) segregated higher 28-day mortality with fair discrimination (AUC ≈ 0.73), supporting serial ECW/TBW as a quantitative “how-much” complement to VExUS/LUS “where/when” endpoints in our case [16].

A contemporary Core Curriculum synthesizes a modern pathophysiology of volume disorders that maps onto our trajectory: in sepsis, endothelial glycocalyx injury converts transvascular exchange into a largely one-way interstitial flux requiring lymphatic clearance; modest positive balances can thus amplify interstitial edema and venous congestion. Rising renal venous pressure lowers the arteriovenous gradient, elevates renal interstitial/tubular pressure within a noncompliant capsule, and triggers neurohumoral activation; pathways that aggravate SA-AKI independent of arterial hypotension and explain dilutional biochemistry in massive FO. Chloride physiology and its impact on renin signaling and diuretic responsiveness further contextualize diuretic resistance in mixed septic–cardiogenic states, reinforcing a phase-specific strategy that pairs imaging-defined congestion (VExUS/LUS) with quantitative BIA targets [17].

Additional sepsis-focused data help situate our approach in SA-AKI with FAS. First, a prospective CRRT cohort identified BIA-derived ECW/TBW ≥ 0.41 as a high-risk stratum for 28-day mortality [16], reinforcing early, measurement-informed de-resuscitation in patients whose FO clearly exceeds such thresholds—like ours. Second, in a randomized strategy trial of SA-AKI on CRRT, a BIA-guided volume-control approach increased UF doses/rates but did not improve 28- or 90-day survival versus conventional care; critically, the achieved “volume accumulation rate” over the first 72 h (cumulative fluid balance divided by baseline BIA-measured overhydration) independently associated with mortality, with >50% denoting markedly worse outcomes—implicating goal achievement rather than tool presence as the determinant [18]. Third, in a multicenter prospective sepsis cohort, VExUS ≥ 2 was uncommon early (18% day 1) and not associated with AKI, mortality, or RRT, nor correlated with CV, thus positioning VExUS as a physiological congestion endpoint rather than a stand-alone prognostic marker in sepsis [8]. In our course, serial VExUS normalization tracked with diuresis and hemodynamic recovery; we therefore used VExUS/LUS to decide when and where to remove fluid and BIA (or ECW/TBW surrogates) to determine how much—operationalizing precision de-resuscitation in FAS.

In sum, contemporary stewardship (salvage–optimization–stabilization–de-escalation) advocates early but judicious resuscitation, vigilant FO prevention, and target-directed decongestion once non-responsiveness is established. Our case operationalizes these principles in mixed shock physiology by coupling VExUS (systemic venous congestion), LUS (pulmonary water), and BIA (whole-body fluid) to individualize UF dose and endpoints when standard markers fail—demonstrating a reproducible bedside pathway for safe, effective decongestion in extreme FO akin to this phenotype [12].

## 4. Conclusions

This case of FAS in SA-AKI with mixed septic–cardiogenic shock and CRS-2 highlights the need for individualized, multimodal de-resuscitation strategies beyond fluid balance charts or central venous pressure.

BIA quantified whole-body fluid excess and set “how-much” UF targets (CBD-ICU concept), while VExUS score and LUS localized and graded congestion to guide “where/when” fluid removal. This approach enabled a safe transition from CVVH to iHDF, with progressive decongestion, diuresis recovery, and renal function stabilization.

Key lessons include the early shift from resuscitation to de-resuscitation once fluid non-responsiveness is evident, using BIA to set quantitative UF targets and monitor decongestion, and employing VExUS/LUS as physiologic endpoints to calibrate intensity and prevent under- or over-decongestion. Dynamic reassessment after each UF step is essential to maintain hemodynamic stability and organ perfusion, with a timely transition from CVVH to intermittent therapy when congestion indices and clinical status permit.

Future research should validate this multimodal approach and its impact on outcomes in SA-AKI and critical care populations.

## Figures and Tables

**Table 1:** Serial bioimpedance analysis (BIA) and venous excess ultrasound score (VExUS) in the index patient during decongestion. Measurements were obtained before and after hemodiafiltration (HDF) sessions and are reported with consistent units. Abbreviations: BIA: bioelectrical impedance analysis; IVC: inferior vena cava; VEXUS: venous excess ultrasound score; POCUS: point-of-care ultrasound; TBW, total body water (L); ECW, extracellular water (L); ICW, intracellular water (L; ICW = TBW − ECW); ECW/TBW, extracellular water as percentage of TBW; PA, phase angle (°) at 50 kHz; FBM, fat body mass (kg); FFM, fat-free mass (kg); SMM, skeletal muscle mass (kg); SMI (IMME), skeletal muscle index = appendicular skeletal muscle mass (ASM)/height^2^ (kg/m^2^); R, resistance (Ω) at 50 kHz; Xc, reactance (Ω) at 50 kHz. HDF, hemodiafiltration. VEXUS score: 0 = no congestion; 1 = mild congestion (dilated IVC but normal venous Doppler); 2 = moderate congestion (abnormal Doppler in one abdominal vein); 3 = severe congestion (abnormal Doppler in ≥2 abdominal veins). LUS: Lung ultrasound.

Date	Measurement	Weight (kg)	TBW (L)	ECW (L)	ICW (L)	ECW/TBW (%)	Phase Angle (°)	FBM (kg)	FFM (kg)	SMM	Resistance (Ω)	VEXUS Score	IVC (Diameter/Collapse)	Hepatic Vein Doppler	Portal Vein Doppler	Renal Vein Doppler	LUS
14/08/2024	BIA 1	168.5	129.3	58.3	71.0	45.1	2.1	0.0	168.5	80.62	192.7	3	28 mm/<10%	Reversal systolic flow	Pulsatility > 50%	Biphasic discontinuous	>3 B-lines bilateral, pleural effusion
15/08/2024	BIA 3	166.6	128.8	57.2	71.6	44.4	2.5	0.0	166.6	82.80	192.3	3	27 mm/<15%	Systolic blunting	Pulsatility ~40%	Biphasic	Diffuse B-lines
16/08/2024	BIA 5	161.6	112.9	50.9	62.0	45.1	2.7	9.38	152.2	72.12	219.8	2	23 mm/20%	Systolic blunting	Mild pulsatility	Continuous flow emerging	Moderate B-lines
18/08/2024	BIA 6	160.3	106.7	48.7	58.0	45.7	2.4	16.99	143.3	67.11	240.6	2	21 mm/30%	Systolic blunting	Mild pulsatility	Continuous	Fewer B-lines, residual effusion
21/08/2024	BIA 9	155.0	85.7	40.1	45.6	46.8	2.4	40.68	114.3	51.77	305.3	1-2	19 mm/40%	Normal systolic-diastolic	Continuous	Continuous	Few scattered B-lines
23/08/2024	BIA 11	150.0	99.2	42.5	56.7	42.8	4.2	16.76	133.2	60.72	270.6	1	18 mm/50%	Normal	Continuous	Continuous	Minimal B-lines
25/08/2024	BIA 13	137.5	71.7	33.5	38.2	46.7	2.7	42.22	95.3	41.12	382.3	1	17 mm/>50%	Normal	Continuous	Continuous	Rare B-lines
27/08/2024	BIA 15	133.0	78.7	36.2	42.5	45.9	2.9	27.80	105.2	47.32	337.5	0–1	16 mm/>50%	Normal	Continuous	Continuous	No B-lines
12/09/2024	BIA 17	112.0	66.7	29.3	37.4	44.0	4.5	22.68	89.3	40.22	431.1	0	15 mm/>50%	Normal	Continuous	Continuous	No B-lines
25/09/2024	BIA 18	102.0	55.5	26.5	29.0	47.7	2.8	28.10	73.9	31.04	500.5	0	14 mm/>50%	Normal	Continuous	Continuous	No B-lines

## Data Availability

No new data were created or analyzed in this study. The data used to support the findings of this study are available from the corresponding author on request (Contact M.C.T., michael.cieza@upch.pe).
